# Mass spectrometry data of metabolomics analysis of Nepenthes pitchers

**DOI:** 10.1016/j.dib.2017.07.068

**Published:** 2017-07-29

**Authors:** Muhammad Aqil Fitri Rosli, Kamalrul Azlan Azizan, Syarul Nataqain Baharum, Hoe-Han Goh

**Affiliations:** Institute of Systems Biology, Universiti Kebangsaan Malaysia, UKM, 43600 Bangi, Selangor, Malaysia

**Keywords:** Carnivorous plant, LC-MS, Metabolomics, Nepenthes, Pitcher

## Abstract

Hybridisation plays a significant role in the evolution and diversification of plants. Hybridisation among Nepenthes species is extensive, either naturally or man-made. To investigate the effects of hybridisation on the chemical compositions, we carried out metabolomics study on pitcher tissue of *Nepenthes ampullaria, Nepenthes rafflesiana* and their hybrid, *Nepenthes × hookeriana*. Pitcher samples were harvested and extracted in methanol:chloroform:water via sonication-assisted extraction before analysed using LC-TOF-MS. MS data were analysed using XCMS online version 2.2.5. This is the first MS data report towards the profiling, identification and comprehensive comparison of metabolites present in Nepenthes species.

## Specifications

**Table:** 

Subject area	Biology
More specific subject area	Metabolomics
Type of data	Analysed MS data
How data was acquired	High resolution mass spectrometry data were acquired from MicrOTOF-Q III (Bruker Daltonic) using an ESI positive ionisation coupled with UltiMate 3000 UHPLC system (Dionex).
Data format	Raw mzXML files and analysed results in .xlsx format
Experimental factors	Metabolites were extracted from the pitcher of *N. ampullaria*, *N. rafflesiana* and their hybrid *N. × hookeriana*
Experimental features	Sonication-assisted extracted samples in methanol:chloroform:water (3:1:1) analysed with a LC-TOF-MS and processed using XCMS online version 2.2.5.
Data source location	Bangi, Malaysia (2°55'11.5''N 101°47'01.4''E)
Data accessibility	Supplementary Table 1 and additional files

## Value of the data

• LC-MS data allow the profiling of metabolites for the first time in the three species of Nepenthes.
• This enables the identification of highly expressed metabolites or biomarkers through comprehensive comparison between the three species.
• Metabolomics analysis provides further understanding on the metabolite expression in hybrid plant of Nepenthes. This dataset can be combined with previous transcriptomics studies [1], [2], [3] to elucidate the biosynthesis pathways of secondary metabolites in Nepenthes species.

## 1 Data

This dataset comprises the acquired MS raw data (mzXML files), and analysed data in MS Excel (.xlsx) file (Supplementary Table 1) generated from XCMS online analysis of MS data from pitcher extracts of *N. ampullaria, N. rafflesiana* and their hybrid *N. × hookeriana*.

## 2 Experimental design, materials and methods

### 2.1 Samples collection

Three lowland *Nepenthes* species, *N. ampullaria*, *N. rafflesiana* and *N.* × *hookeriana* were sampled from the experimental terrace of Universiti Kebangsaan Malaysia (2°55'12.7"N, 101°46'59.7"E). Pitcher tissues were harvested 7 days after pitcher opening when the pitchers achieved fully functionality [4]. The pitcher fluids were emptied and rinsed with sterile deionised water before immediately frozen in liquid nitrogen and stored at −80 °C.

### 2.2 Phytochemical extraction

Each sample was crushed and ground until fine powder before lyophilised for 48 h. Extraction from the dried powder was performed according to [5] with slight modifications. Dried powder samples (10 mg) were extracted with 200 µL of methanol:chloroform:water (3:1:1). Samples were vortexed, sonicated at room temperature for 15 min, vortexed again and then centrifuged at 10,000*g* for 10 min. Filtered extracts through a 0.22 µm PTFE membrane were stored at −80 °C.

### 2.3 Liquid Chromatography-Mass Spectrometry (LC-MS) analysis

The chromatographic separation was performed on Thermo Scientific C18 column (Acclaim^TM^ Polar Advantage II, 3×150 mm, 3 µm particle size) on an UltiMate 3000 UHPLC system (Dionex). The gradient elution was performed at 0.4 mL/min flow rate at 40 °C using (A) water containing 0.1% formic acid, and (B) 100% acetonitrile with 22 min total run time. The gradient started at 5% solvent B for 3 min (0–3 min), then the gradient increased to 80% solvent B for 7 min (3–10 min) and maintained at 80% solvent B for 5 min (10–15 min). Finally, the gradient returned to 5% solvent B in 7 min (15–22 min).

High resolution MS was carried out using a MicrOTOF-Q III (Bruker Daltonic) using an ESI positive ionisation with the following settings: capillary voltage at 4500 V, nebuliser pressure at 1.2 bar and drying gas flow at 8 L/min with the source temperature at 200 °C and *m*/*z* range from 50 to 1000 Da.

### 2.4 Mass spectrometry data handling

The acquired MS raw data were converted to the mzXML file format by Bruker Compass DataAnalysisViewer version 4.2. The converted data were processed by XCMS online software package [6] with *Arabidopsis thaliana* selected as the bio-source and other default settings to carry out feature detection, peak alignment, retention time correction, statistical analysis, annotation and identification.

## References

[1] Zulkapli M.M., Rosli M.A.F., Salleh F.I.M., Mohd Noor N., Aizat W.M., Goh H.H. (2017). Iso-Seq analysis of *Nepenthes ampullaria, Nepenthes rafflesiana* and *Nepenthes × hookeriana* for hybridisation study in pitcher plants. Genom. Data.

[2] Wan Zakaria W.N.A., Loke K.K., Goh H.H., Mohd Noor N. (2016). RNA-seq analysis for plant carnivory gene discovery in *Nepenthes × ventrata*. Genom. Data.

[3] Wan Zakaria W.N.A., Loke K.K., Zulkapli M.M., Mohd Salleh F.I., Goh H.H., Mohd Noor N. (2016). RNA-seq analysis of *Nepenthes ampullaria*. Front. Plant Sci..

[4] Bauer U., Willmes C., Federle W. (2009). Effect of pitcher age on trapping efficiency and natural prey capture in carnivorous *Nepenthes rafflesiana* plants. Ann. Bot..

[5] Goh H.H., Khairudin K., Sukiran N.A., Normah M.N., Baharum S.N. (2016). Metabolite profiling reveals temperature effects on the VOCs and flavonoids of different plant populations. Plant Biol..

[6] Tautenhahn R., Patti G.J., Rinehart D., Siuzdak G. (2012). XCMS online: a web-based platform to process untargeted metabolomic data. Anal. Chem..

